# (Swiss) GraphoLearn: an app-based tool to support beginning readers

**DOI:** 10.1186/s41039-020-0125-0

**Published:** 2020-02-28

**Authors:** Hannah Mehringer, Gorka Fraga-González, Georgette Pleisch, Martina Röthlisberger, Franziska Aepli, Vera Keller, Iliana I. Karipidis, Silvia Brem

**Affiliations:** 1 Department of Child and Adolescent Psychiatry and Psychotherapy, University Hospital of Psychiatry, University of Zurich, Neumünsterallee 9, 8032 Zurich, Switzerland; 2 Neuroscience Center Zurich, University of Zurich and ETH Zurich, Zurich, Switzerland; 3 MR-Center of the University Hospital of Psychiatry and the Department of Child and Adolescent Psychiatry and Psychotherapy, University of Zurich, Zurich, Switzerland

**Keywords:** GraphoLearn, Beginning readers, Children at risk for developmental dyslexia, Computer-based training, Grapheme-phoneme learning

## Abstract

We assessed the Swiss-German version of GraphoLearn, a computer game designed to support reading by training grapheme-phoneme correspondences. A group of 34 children at risk for dyslexia trained three times a week during 14 weeks, on top of their standard school instruction. The sample was divided into two groups of 18 and 16 children, who started training at either the middle or the end of first grade. We found beneficial training effects in pseudoword reading in both training groups and for rapid automatized naming skills in the group that trained earlier. Our results suggest that both the efficiency in phonological decoding and rapid access to verbal representations are susceptible to facilitation by GraphoLearn. These findings confirm the utility of the training software as a tool to support school instruction and reading-related abilities in beginning readers. We discuss ideas to improve the content and outcomes of future versions of the training software.

## Introduction

Reading is essential in our literate society and impairments in learning how to read can have adverse psychosocial consequences. This is the case in individuals diagnosed with dyslexia, which is among the most common neurodevelopmental disorders with a prevalence of 3–8% (Galuschka, Ise, Krick, & Schulte-Körne, 2014) and various genetic and environmental factors involved in its etiology (Carrion-Castillo, Franke, & Fisher, 2013). Affected children typically show much slower and inaccurate reading, have great difficulties in spelling and often show insufficient reading comprehension, especially when reading long sentences. Importantly, dyslexia is highly persistent and can lead to severe impairments throughout school age and even in adulthood (Fraga-González, Karipidis, & Tijms, Fraga-González, Karipidis, & Tijms, 2018). Moreover, the negative impact in academic performance often results in pronounced fears of failure and a negative self-concept in impaired readers. In addition, comorbidity of dyslexia with other disorders such as anxiety and attention deficit disorders is relatively common.

Dyslexia runs in families and children with a dyslexic parent or sibling have a high risk (30 to 65%) of being affected too, and should be carefully monitored during reading acquisition (Pennington & Lefly, 2001; Scarborough, 1991). Children at risk for dyslexia need to be identified as early as possible and be supported effectively at the beginning of reading instruction (Torgesen, 2004). Besides early risk detection, providing these children with additional support to school practice can be an important intermediate step to prevent the development of severe reading impairments and the need for costly specialized treatments. Intervention and training studies have suggested that a few elements are particularly important for facilitating fluent reading skill acquisition and develop efficient support tools.

Diverse intervention approaches have been proposed to improve reading and spelling skills of children with dyslexia. The phonics instruction mainly consists of a systematic instruction of grapheme-phoneme correspondences including decoding strategies and exercises with blending, word segmentation, elimination or adding phonemes to words which is an indispensable prerequisite for learning to read (Galuschka et al., 2014; Ziegler & Goswami, 2005). Learning such correspondences is crucial to transition from a slow grapheme by grapheme conversion (phonological or indirect route) to a fast, automatic access (direct route) to a word meaning while reading (Ehri, 2005). Phonics instruction is the most frequently investigated approach and the only one that was confirmed as effective in a meta-analysis of randomized controlled trials (Galuschka et al., 2014). Other interventions focused on auditory or visual processing or general functions did not show significant improvements in reading or spelling in children with dyslexia (Ise, Engel, & Schulte-Körne, 2012).

Computerized interventions on grapheme-phoneme learning are gaining support and relevance in research. In a clinical setting, a computer-based intervention combining explicit instruction and intensive practice on letter-speech sound correspondences was effective in helping dyslexic children to obtain a level of reading accuracy and spelling comparable to that of normal readers (Tijms, 2011). In addition, performance in a computer-based training on implicit learning of artificial letter-speech sound associations was related to individual differences in reading and spelling skills, as well as to intervention response in dyslexic readers (Aravena, Tijms, Snellings, & van der Molen, 2016, 2017), while performance of prereaders in a similar artificial-letter training was associated with their early reading fluency skills (Karipidis et al., 2018). In the last few years, this type of computerized programs are increasingly being used (Blumberg & Fisch, 2013) to supplement traditional reading instruction. From an educational perspective, their main advantages are the potential to motivate children to learn, the relatively easy availability compared with a skilled personal trainer and the possibility to individualize programs (Maracuso & Walker, 2008). These tools can make training available at any time so that children can practice at their own pace to support word recognition skills and eventually fluent text reading. Importantly, they have also been shown to be useful as support tools for populations at risk (e.g., Saine, Lerkkanen, Ahonen, Tolvanen, & Lyytinen, 2011).

The current study evaluates GraphoLearn, a computer-based tool for reading instruction that through phonics training explicitly introduces and trains grapheme-phoneme correspondences of increasing complexity (Richardson & Lyytinen, 2014). In short, GraphoLearn provides a computerized training of a series of key elements shown to be important for fluent reading acquisition, with an emphasis on grapheme-phoneme correspondences but extending the training exercises from letters to word and short sentence reading. The software offers a game-like environment which is engaging for children of different ages and reading levels. The purpose of the game is not to replace initial reading instruction or remediate dyslexia. Rather, it is designed to provide additional practice to children, allowing them to play on their own and supporting their reading skills according to their individual skill level. Because of this, it is well suited to be utilized as a preventive tool for those children at risk of becoming impaired without additional support outside school. The GraphoLearn environment was originally developed in Finnish (Lyytinen, Erskine, Hämäläine, Torppa, & Ronimus, 2015; Richardson & Lyytinen, 2014) and has been adapted for several other languages. In school-children of different backgrounds, GraphoLearn has mainly improved decoding and spelling skills (Jere-Folotiya et al., 2014; Kyle, Kujala, Richardson, Lyytinen, & Goswami, 2013; Ojanen, Kujala, Richardson, & Lyytinen, 2013; Saine et al., 2011) or letter knowledge (Patel, Torppa, Aro, Richardson, & Lyytinen, 2018; Saine et al., 2011; Kamykowska, Haman, Latvala, Richardson, & Lyytinen, 2013), but in some reports, the significant improvements extended to reading skills of young poor readers (Ruiz et al., 2017; Saine, Lerkkanen, Ahonen, Tolvanen, & Lyytinen, 2010). GraphoLearn has also been shown to improve neural specialization to letters in prereaders, reflected in a stronger response to print (letters and letter-like false fonts) in the left occipito-temporal cortex, also known as the visual word-form system (Brem et al., 2010). Thus, previous assessments of the GraphoLearn show beneficial effects that seem to differ in the particular literacy subskills improved by training. Orthographic transparency is likely to be a crucial factor to explain these differences, since acquiring fluent decoding is more complicated in opaque orthographies like English, with irregular and complex grapheme-phoneme mappings, as compared to more transparent or semitransparent orthographies like German (Seymour, Aro, & Erskine, 2003).

In the present study, GraphoLearn was adapted for the German-speaking part of Switzerland. We aimed to provide a supportive tool that beginning readers could use independently to improve their reading skills. To evaluate its applicability and effectivity, we conducted a small-scale study following children at risk for dyslexia in first and second grade, as they constitute the population that could benefit the most from the training software. We first examined how users performed in the GraphoLearn training. Then, in order to address our main aim, we assessed improvements in reading fluency (word and pseudoword reading), phonological processing and rapid automatized naming (alphanumeric and non-alphanumeric) tasks. We expect that intensive training with GraphoLearn helps beginning readers to enhance decoding, expand their orthographic lexicon and to correspondingly improve reading skills.

## Materials and methods

### Participants

The current study focuses on a subsample of 34 children that were classified as below-average readers from an initial sample of 50 children in kindergarten and first grade at risk for dyslexia recruited for a longitudinal neuroimaging study (Karipidis et al., 2017, 2018). The complete sample characteristics are described in Table 1, and the group assignment is described in the next section. Recruitment was done via advertisements and brochures that were distributed at schools. All children were (Swiss) German native speakers and had an average or above-average intelligence estimate. Exclusion criteria were physical, neurological or psychiatric disorders apart from the often-comorbid attention deficit hyperactivity disorder (ADHD). Two children had a diagnosis of ADHD at the time of participation but received either no medication or the medication was discontinued 48 h before the tests. All children were at familial risk for dyslexia (*n* = 48) or had a diagnosis of delayed speech development (*n* = 2). Parents filled out the Adult Reading History Questionnaire (ARHQ) (Lefly & Pennington, 2000), a self-assessment questionnaire in which a score > 0.30 indicates a history of reading disability. Familial risk was present when at least one parent scored > 0.30 in the ARHQ (*n* = 45) or a sibling had reading problems (*n* = 3). The research protocol was approved by the local ethics committee and carried out in accordance with the recommendations of the ordinance on human research. All parents gave written informed consent in accordance with the Declaration of Helsinki.

**Table 1:** Sample characteristics and baseline comparisons at T1 (middle of grade 1)

	Training groups		Control	Contrasts						
	Group 1 *n* = 18	Group 2* n = 16	Good readers *n* = 16	TG1 : CG		TG2 : CG		TG1 : TG2		
	M (SD)	M (SD)	M (SD)	*t*	*p* value	*t*	*p* value	*t*	*p* value	
Sex ratio (f:m)	10:8	5:11	11:5							
Age	7.22 (0.30)	7.34 (0.38)	7.24 (0.29)	−0.21	.832	0.83	.414	−1.03	.310	
IQ (CFT-1)^a^	99.78 (7.04)	99.50 (12.77)	103.93 (11.99)	−1.24	.225	−0.99	.328	0.08	.937	
Reading tests										
SLRT-II^b^										
Words	17.61 (14.53)	23.43 (19.34)	51.91 (35.73)	−**3.74**	**.001**	−**2.73**	**.011**	−0.99	.331	
Pseudowords	13.11 (13.29)	15.00 (16.50)	46.44 (33.53)	−**3.89**	**.000**	−**3.28**	**.003**	−0.36	.718	
Upper-case word reading	11.33 (6.09)	13.87 (6.49)	25.50 (17.83)	−**3.18**	**.003**	−**2.38**	**.024**	−1.15	.257	
Local reading test										
Words	12.56 (9.22)	13.93 (5.85)	20.13 (7.43)	−**2.56**	**.015**	−**2.54**	**.017**	−0.50	.620	
Pseudowords	8.83 (8.27)	11.93 (9.03)	19.07 (7.48)	−**3.73**	**.001**	−**2.36**	**.026**	−1.03	.312	
Phonological processing										
TEPHOBE										
Rhyme	4.72 (1.45)	5.00 (1.56)	5.56 (1.03)	−*1.93*	*.063*	−1.19	.243	−0.53	.600	
Initial sound categorization	4.89 (2.03)	5.80 (1.78)	6.38 (1.15)	−**2.59**	**.014**	−1.08	.291	−1.36	.184	
BAKO										
Phoneme deletion	2.72 (2.16)	2.20 (2.11)	4.13 (2.03)	−*1.94*	*.061*	−**2.59**	**.015**	0.70	.490	
Pseudoword segmentation	3.11 (1.28)	2.67 (1.95)	3.88 (1.46)	−1.63	.113	−*1.96*	*.059*	0.80	.438	
Vowel substitution	5.06 (2.88)	4.00 (2.56)	6.50 (1.59)	−*1.78*	*.085*	−**3.29**	**.003**	1.10	.279	
Rapid automatized naming										
Letters**	0.89 (0.30)	1.06 (0.28)	1.42 (0.45)	−**4.10**	**.000**	−**2.59**	**.015**	−1.65	.109	
Numbers**	0.90 (0.23)	1.00 (0.16)	1.31 (0.38)	−**3.81**	**.001**	−**2.86**	**.008**	−1.28	.209	
Colors	0.60 (0.20)	0.61 (0.17)	0.84 (0.31)	−**2.71**	**.011**	−**2.54**	**.017**	−0.15	.883	
Objects	0.65 (0.13)	0.66 (0.12)	0.83 (0.17)	−**4.10**	**.000**	−**3.16**	**.004**	−0.06	.953	

^*^Data missing for 1 participant at T1 (N_T1_ = 15)

^**^Data missing for 2 participants for group 2 (N_T1_ = 14)

^a^Measured at T3

^b^Percentile score

TG1 = Group 1; TG2 = Group 2; *CG* Control group; bold text indicates significant results (*p* < 0.05); italic text represents results at trend level

### Study design and group assignment

In the middle of first grade (T1), after around 5 months of formal reading instruction at school, participants were classified as below-average and typical readers based on their initial reading fluency and from an additional measure of phonological precursor skills that was available for 41 children from an earlier assessment in preschool. They were classified as below-average readers if they performed below percentile 40 in three 1-min reading fluency tests (see Behavioral Assessment section) or below percentile 30 in one test and below percentile 50 in the other two tests. Children with low phonological skills as prereaders were classified as below-average readers if they scored below percentile 50 in all reading tests. We considered low phonological skills scores below percentile 30 in phonological awareness and/or rapid automatized naming (RAN), which are strong predictors of developmental dyslexia (Furnes & Samuelsson, 2011; Rückert Mandu, Kunze, Schiller, & Schulte-Körne, 2010). The focus of our analyses is on the 34 below-average readers in this sample. All remaining children (*n* = 16) were classified as typical readers and not included in the analyses except for the baseline assessment comparison (T1).

After the tests in the middle of first grade (T1), all participants were tested after training/waiting control period at the end of first grade (T2) and at the beginning of second grade (T3). A follow-up was done for some tests in the middle of second grade (T4). In a single-blinded randomized cross-over training design, below-average readers were divided into two training groups matched by age and performance in T1 reading tests. Group 1 (*N* = 18) trained from T1 to T2, while group 2 (*N* = 16)^1^ acted as a waiting control group during that period and trained from T2 to T3 instead. Both groups trained for around 14 weeks and approximately 3 to 4 months elapsed between assessments (T1–T2, T2–T3, T3–T4); the mean (SD) number of months elapsed between assessments in training group 1 were 3.28 (0.67), 3.39 (0.50) and 4.33 (2.63). For the training group 2, they were 3.07 (0.26), 3.69 (0.48), and 5.06 (3.96). The trained psychologists performing the behavioral assessments were blinded about group assignment, that is, they did not know whether the participant had trained with the game, and children were instructed not to talk to the experimenters about the training.

### Behavioral assessments

Participants performed a series of tests on reading, phonological awareness, and alphanumeric/non-alphanumeric rapid automatized naming. Reading fluency was measured with the 1-min reading tests for words and pseudowords included in the *Salzburger Lese- und Rechtschreibtest* (SLRT-II; Moll & Landerl, 2014). In these tests, fluency is measured by the number of words or pseudowords correctly read within 1 min from a list of 156 items presented in eight columns. The SLRT-II word and pseudoword reading tests are available in two forms (A and B) with normed test scores starting from the end of first grade. Form A was used to assess reading at T1, T2 and T3, but form B was used for the follow-up at T4. Therefore, our analysis of training effects on SLRT-II raw scores only includes the time points T1 to T3 to avoid biases related to differences in the items of the lists. Raw scores were used in the group comparisons since standardized test scores were not available for all time points. Additionally, we created a list of upper-case words to test reading fluency of easy words only for the baseline comparisons and group assignment. For these purposes, we computed local norms for the upper-case word reading fluency test, as well as SLRT-II word and pseudoword reading fluency tests. This was done by assessing the reading data of a representative group (*n* = 75) of German-speaking children in and around the Canton of Zurich. The upper-case word reading test was not used to test training effects given its low difficulty. Moreover, we designed an additional measure of reading accuracy to incorporate specific items that were trained with GraphoLearn. The local reading lists of words and pseudowords included items from the GraphoLearn training and items not presented during the training. They were not time-limited, included 30 words and 30 pseudowords and they were presented at every time point. An accuracy measure of correctly read words and pseudowords was computed.

Phonological decoding was measured at T1 to T3 with subtests from two standardized tests. The tests were not used at T4 as they were designed for children up to first grade. We used the rhyme and initial sound categorization subtests from Test zur Erfassung der phonologischen Bewusstheit und der Benennungsgeschwindigkeit (TEPHOBE; Mayer, 2013). In the rhyme subtest, children must select two rhyming words out of four aurally presented words, whereas in the initial sound categorization subtest, two out of four words starting with the same speech sound have to be selected. To control for working memory biases, children are provided with pictures of the aurally presented items. Both subtests consist of seven trials. In addition, we used several subtests from the Basiskompetenzen für Lese-Rechtschreibleistungen (BAKO; Stock, Marx, & Schneider, 2003). In the phoneme deletion subtest, the children have to detect and delete the initial speech sound of a word or pseudoword and pronounce it aloud without that sound across seven trials. In the pseudoword segmentation subtest (eight trials), the children have to segment (aurally presented) pseudowords by vocalizing each phoneme separately while moving tokens representing the phonemes towards the experimenter (e.g., ‘*skop*’ → /*s/-/k/-/o/-/p/*). In the vowel substitution subtest, children repeat the presented items replacing all instances of the vowel ‘A’ with ‘I’ (e.g., ‘*Hans*’ → ‘*Hins*’). There are 12 trials of increasing complexity, including words with more than one vowel. The number of correct trials was used as the score in these tests.

Rapid automatized naming (RAN) requires rapid and automatized visual identification of items and access to the corresponding verbal representation in long-term memory. We tested RAN at all time points using the subtests in TEPHOBE (Mayer, 2013), i.e., RAN of letters, numbers, colors, and objects. Each subtest has 10 practice and 50 test items (listed in 10 lines of 5 items each). The children are asked to name all items subsequently as fast as possible. The scores indicate the number of items read per second. To prevent potential bias in cases with red-green blindness in the RAN of colors subtest (which includes green, red, blue, brown, and yellow colors), we also tested a version with the colors gray, white, blue, brown, and yellow.

Finally, nonverbal IQ was assessed with the revised short version of the Cultural Fair Intelligence Test (CFT 1-R; Weiss & Osterland, 2013) at T3. The test consists of six subtests with durations between 70 s and 3 min. The first three subtests measure visual attention and speed of processing and the remaining subtests measure logical reasoning.

### GraphoLearn training and procedure

GraphoLearn, previously known as GraphoGame (http://info.grapholearn.com), is a computer-based tool for reading instruction that explicitly introduces and trains grapheme-phoneme correspondences (Richardson & Lyytinen, 2014). The current GraphoLearn is based on a previous version of the same computer game that led to improvements in letter knowledge and beginning visual specialization in the brain of preschool children after training grapheme-phoneme correspondences (Brem et al., 2010; Brem et al., 2013). The current GraphoLearn is adjusted to the semi-transparent orthography of Standard German and takes into account the differences between spoken Swiss and Standard German in the pronunciation of several phonemes. The instructions and the auditory stimuli were spoken by two professional speakers, one female and one male, in the Swiss variety of Standard German. The game consists of 18 thematic streams of increasing difficulty, each containing 27 to 50 levels. It starts with easy, transparent phonemes with a one-to-one phoneme-grapheme relationship in streams 1 to 6, then introduces semi-transparent and opaque correspondences in streams 7 to 11 and 12 to 18 (Röthlisberger et al., 2015). Auditory stimuli of increasing complexity are presented, from single letters to syllables, word parts, words, pseudowords and short sentences.

The player is guided through the training by an avatar of his or her choice. In most levels, the player is instructed to click on the grapheme(s) that correspond to the presented auditory information. The target grapheme is presented among one to nine distractors; their number is adaptive as it decreases after incorrect answers to avoid excessive frustration. The player receives immediate feedback on whether the answer was correct (green highlight) or not (red). Correct answers lead to the next trial and a reward in the form of coins that can be used to equip the avatar with accessories. Each level is repeated up to five times when children fail in reaching the expected accuracy threshold. Additional training forms include rhyming tasks, word building, and sentence building.

All children received a laptop, a child-friendly computer mouse, and high-quality over-ear headphones to use at home during the training. They were instructed to train three times a week for 20 min over 14 weeks. We estimated that a session would result in around 15 min of effective training; thus, effective training time was expected to add up to around 10 h. Parents were informed that GraphoLearn was not intended to replace school learning, but only as a support tool for reading. The training data was regularly uploaded into a university server to monitor progress and if children did not play for at least 1 h per week, parents were contacted by the project team to encourage further training. On top of the rewards in the game, children received a sticker for every hour played (ten stickers resulted in a small present).

### Statistical analyses

We first examined potential group differences in baseline reading skills and training performance. Then, as our main analysis, we examined training effects in reading fluency, phonological processing and rapid automatized naming by comparing the training periods to waiting control periods. All analyses were done using IBM SPSS Statistics 23 software and the Statistical Analysis Software (SAS, Version 9.4). Plots were created using ‘ggplot2’ package for R. Corrected *p* values ≤ .05 were considered significant and *p* values ≤ .1 are reported as trends. Finally, we calculated effect sizes of the most relevant effects using Cohen’s *d*_*z*_ (computed as the mean difference between two time points divided by the standard deviation of this difference).

#### Baseline comparisons and training performance

The group differences in baseline reading measures and training performance were examined with independent sample *t* tests.

#### Training effects on reading, phonology and RAN

The main analysis of GraphoLearn training effects was performed using linear mixed models on raw scores (see Behavioral Assessments section) with the fixed factor time (T1, T2, T3, and T4 when available) and group (training groups 1 and 2). The model included first all-time points available. As an additional analysis, we then applied the model including only the time points of the first (T1–T2) and second (T2–T3) training periods to corroborate training-specific gains without the potential bias of including a third-time point in the analysis. Significant interactions in the models were followed by post hoc tests with *p* values corrected for multiple comparisons (*p*_corr_) using the Tukey-Kramer method. The normality of raw scores was assessed by inspection of q-q plots and a log-transformation was applied to those scores that were not normally distributed. We excluded outliers based on the standardized residuals of the linear mixed models (cases deviating more than 2.5 standard deviations from the mean).

## Results

### Baseline comparisons

The training groups did not differ in their reading performance and both showed poorer reading skills than the typical reader group from the same initial sample of at-risk children. All groups had comparable age at T1 and IQs at T3. The baseline comparisons are shown in Table 1.

### Training exposure and performance

Participants from the training group 2 played significantly more levels than training group 1 (group 1259.50 ± 109.29 levels; group 2368.94 ± 114.32 levels; *t* (32) = −1.37; *p* = .008) and there was a trend for faster working speed in group 2 (group 10.425 ± 0.12; group 2, 0.497 ± 0.12; *t* (32) = −1.75; *p* = .090). The groups did not significantly differ in exposure time, *p* = .181. The difference in the number of levels could be explained by faster progress of group 2 relative to group 1 in the initial levels of the training due to their more advanced reading stage at the start of the training interval. The group comparisons and descriptive statistics of the mean training interval in days, duration of the training in minutes (exposure time), number of levels played and working speed (levels per minute) are displayed in Additional file 1: Table S1.

### Training effects

Our main analysis consisted of a linear mixed model to examine interactions between the factors group and time and subsequent *t* tests for each time period. For brevity, we do not report the main effects of the between-subject factor group at each individual time point since they did not reach statistical significance in any of the outcome measures, all *ps* > .221.

#### Reading fluency

Our primary measures were those related to fluency in word and pseudoword reading from the SLRT-II as a main test for classification of below-average readers. These measures were complemented by two local lists of words and pseudowords which also included items of the training.

#### Word reading

##### SLRT II—words

We compared the raw scores in the SLRT-II 1-min word reading test between T1, T2, and T3. The results for each group and time point are shown in Additional file 1: Figure S1 (see scores in Table 2). The analysis revealed a significant main effect of time (*F*(2,58) = 63.73; *p* < .001) suggesting overall improvements across time points, and marginally non-significant interaction between time and group (*F*(2,58) = 2.99; *p* = .058).

**Table 2:** Descriptive statistics showing mean (SD) of behavioral assessments per group across time points

	Group 1 *n* = 18				Group2* n = 16			
	T1	T2	T3	T4	T1	T2	T3	T4
Reading								
SLRT-II^a^								
Words	5.28 (3.91)	10.39 (6.67)	16.94 (10.76)		6.67 (4.37)	12.88 (9.70)	19.31 (9.58)	
Pseudowords	9.28 (6.42)	14.33 (6.02)	17.44 (7.16)		10.27 (6.18)	12.38 (7.16)	17.88 (8.49)	
Local reading test								
Words	12.56 (9.22)	15.67 (7.39)	19.67 (6.61)	23.33 (4.66)	13.93(5.85)	15.87 (8.57)	20.50 (4.84)	23.25 (4.89)
Pseudowords	8.83 (8.27)	13.11 (7.78)	15.39 (8.19)	18.94 (5.71)	11.93 (9.03)	10.44 (7.82)	16.00 (6.46)	16.69 (7.81)
Phonological processing								
TEPHOBE								
Rhyme	4.72 (1.45)	5.39 (1.20)	5.83 (1.15)	–	5.00 (1.56)	4.88 (1.09)	5.31 (1.25)	–
Initial sound categorization	4.89 (2.03)	6.00 (1.14)	6.33 (1.14)	–	5.80 (1.78)	6.19 (1.22)	6.56 (0.73)	–
BAKO								
Phoneme deletion**	2.72 (2.16)	3.31 (2.12)	3.61 (1.85)	–	2.20 (2.11)	2.69 (1.25)	3.12 (1.67)	–
Pseudoword Segmentation	3.11 (1.28)	3.72 (1.56)	3.67 (1.61)	–	2.67 (1.95)	3.06 (1.29)	3.38 (0.81)	–
Vowel substitution	5.06 (2.88)	5.78 (1.59)	6.22 (1.63)	–	4.00 (2.56)	4.88 (2.42)	5.75 (1.84)	–
Rapid automatized naming								
Letters***	0.89 (0.30)	1.21 (0.29)	1.17 (0.33)	1.33 (0.33)	1.06 (0.28)	1.20 (0.44)	1.22 (0.42)	1.38 (0.50)
Numbers***	0.90 (0.23)	1.07 (0.25)	1.06 (0.37)	1.23 (0.43)	1.00 (0.16)	1.00 (0.23)	1.10 (0.21)	1.22 (0.23)
Colors	0.60 (0.20)	0.69(0.20)	0.67 (0.21)	0.74 (0.22)	0.61 (0.17)	0.60(0.18)	0.68 (0.14)	0.75 (0.15)
Objects	0.65 (0.13)	0.75(0.17)	0.76 (0.19)	0.81 (0. 19)	0.66 (0.12)	0.67 (0.15)	0.74 (0.15)	0. 82 (0. 11)

*Data missing for 1 participant at T1 (N_T1_ = 15); **Data missing for 3 participants at T2 in group 2 (N_T2_ = 13); ***Data missing for 2 participants at T1 in group 2 (N_T2_ = 14); ^a^SLRT-II scores from T4 not included in main analysis as they were obtained in different lists of items

To further examine this trend, we first looked at learning trajectories with separate post hoc *t* tests per group. The analysis showed that group 1 significantly improved word reading during their training period, (*t* (58) = −4.35; *p* < .001, *p*_corr_ = .001) but not afterwards, *p*_corr_ = .173. Group 2, on the other hand, showed significant improvements between T1 and T2 (*t*(58) = −4.63; *p* < .001, *p*_corr_ < .001) as well as during their training period (*t*(58) = −5.16; *p* < .001, *p*_corr_ < .001). Further examination of these effects shows large effect sizes and comparable delta scores associated with gains in both the training and waiting control periods overall (Cohen’s *d*_*z*_ of 1.34 and 1.01 respectively). Thus, gains in word reading could not be attributed solely to additional training.

Additionally, we performed tests at the first and second time periods (T1–T2 and T2–T3, respectively) to investigate the time and group interactions that would indicate training-specific effects, without the potential confound of including additional time periods in the model. This interaction was not statistically significant in any of the two time periods, *ps >* 0.307, and the main effect of time was significant in both T1–T2 (*F* = 31.29, *p* < 0.001) and T2–T3 (*F* = 59.62, *p* < 0.001) periods. This further corroborates that gains in SLRT word reading were mainly related to schooling.

##### Local reading test: word list

As an additional reading measure, we presented a local list of words at T1 to T4 (group means are presented i Additional file 1: Figure S2). The linear mixed model analysis showed a main effect of time (*F*(3,87) = 65.92; *p* < .001) and a trend for interaction between time and group (*F*(3,87) = 2.18; *p* = .096). In the post hoc *t* tests, group 1 did not significantly improve during their training period, *p*_corr_ = .625 but showed a significant improvement in the later periods T2–T3, (*t*(87) = −3.63; *p* < .001, *p*_corr_ = .011) and T3–T4 (*t*(87) = −5.19; *p* < .001, *p*_corr_ < .001). Training group 2 only showed significant improvement during their training period, i.e., T2 to T3 (*t*(87) = −4.62; *p* < .001, *p*_corr_ < .001) and not in the other periods, *ps*_corr_ > .392. As anticipated by these results, no significant interactions between time and group in the additional tests at each time period were found, *ps* > 0.294, although the main effect of time was significant in both periods (T1–T2; *F* = 17.79, *p* < 0.001 and T2–T3; *F* = 34.05, *p* < .001).

#### Pseudoword reading

##### SLRT II—pseudowords

**Fig. 1:**
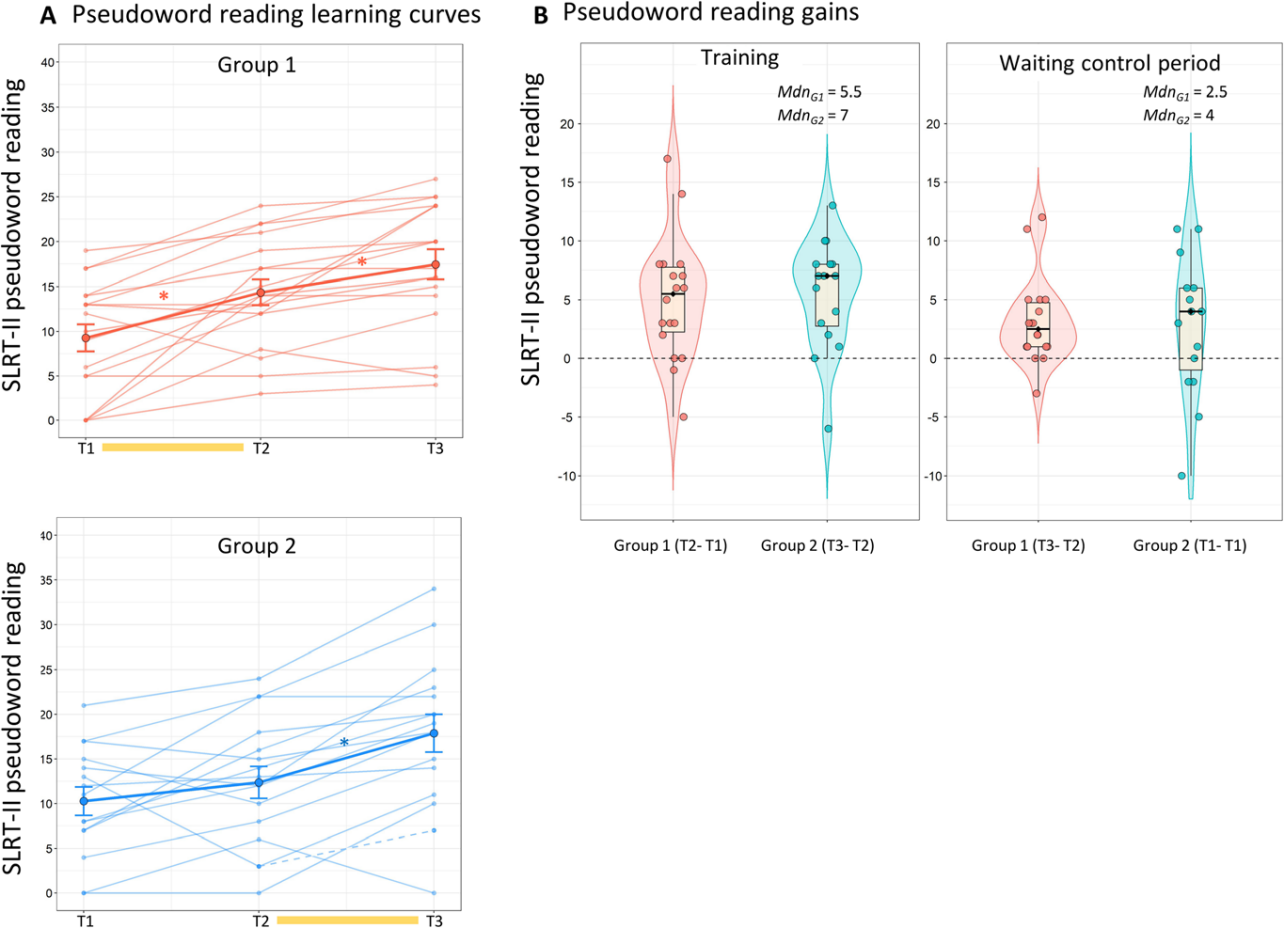
Results for the SLRT-II pseudoword reading task. **a** Line plots show individual scores and group means across the 3-time points for group 1 (top) and group 2 (bottom). The training period for each group is indicated by a yellow bar in the x-axis. Dashed lines indicate children for which the data of one test time was missing, error bars indicate SEM and asterisks indicate significant *t* tests at *p*corr < 0.05. **b** Violin and box plots show delta scores for each group during the training period (left) and waiting control period (right). Black horizontal bar indicates the median. Data from group 1 are shown in red and data from group 2 in blue

The linear mixed model analysis with the pseudoword reading test of the SLRT-II revealed a significant main effect of time, *F*(2,62) = 49.30; *p* < .001, indicating overall better performance over time (see Table 2 and Fig. 1a). The interaction between time and group was marginally significant (*F*(2,62) = 2.99; *p* = .058). Post hoc *t* tests per group showed a significant improvement in group 1 during their training period (*t*(62) = −4.11; *p* < .001, *p*_corr_ = .002) and after training (*t*(62) = −3.48; *p* = .001, *p*_corr_ = .011). Group 2 showed a significant improvement only in the training period, i.e., between T2 and T3 (*t*(62) = −6.43; *p* < .001, *p*_corr_ < .001), and no significant changes in the preceding period (T1–T2), *p*_corr_ = .331. To further qualify the differences between training and waiting periods in the groups, we examined the effect sizes of the gains in pseudoword reading (see delta score distributions in Fig. 1b). In the pooled sample, we found large effects associated with gains after training and only medium-sized effects in the period without training, Cohen’s *d*_*z*_ 1.07 and 0.62, respectively. We further examined the different patterns of gains in the two groups separately. In group 1, effect sizes for training and school period were 0.96 and 0.85 and in group 2 they were 1.18 and 0.47, respectively. Gains in group 2 during the waiting control period were not statistically significant and thus the small effect size was expected. When examining individual differences in improvement, delta scores suggest a relatively low rate of poor responders to training in our sample. After training, 27.78 % of participants in group 1 were below the group’s median gains during their waiting control period (Mdn = 2.5), while in group 2, that was the case for 31.25 % of participants (Mdn = 4). Note that the learning rate in the waiting control period of group 2 shows four children with a decline in performance over time and a more spread distribution than group 1 at that period and both groups during the training period (see Fig. 1b right panel).

The additional model including only two time points in the first and second time periods, revealed a significant interaction between time and group at the T2–T3 period (*F* = 6.34, *p* = .017), confirming more pronounced gains in the group that received training at that period (group 2). This interaction, however, did not reach statistical significance in the earlier time period, *p* = .197. The main effect of time was significant in both periods (T1–T2; *F* = 15.21, *p* < 0.001 and T2–T3; *F* = 54.61, *p* < .001).

##### Local reading test: pseudoword list

The linear mixed model analysis revealed a significant effect of time (*F*(3,92) = 21.23; *p* < .001) and a significant interaction between time and group (*F*(3,92) = 2.87; *p* = .040; see Additional file 1: Figure S2). In the post hoc *t* tests, improvements in group 1 did not reach statistical significance during the training or the waiting control period, *ps*_corr_ > .267, but it approached trend levels in the last follow-up period (*t*(92) = −2.80; *p* = .006, *p*_corr_ = .108). Training group 2 showed significant improvement only during the training (T2–T3; *t*(92) = −3.80; *p* < .001 , *p*_corr_ < .001) but not in the other periods, *ps*_corr_ > .997. Importantly, we should note that the T1–T2 training effect in group 1 reached trend levels (*t*(56) = −2.86; *p* = .006, *p*_corr_ = .062) after excluding the T4 follow-up (interaction time by group, *F*(2,56) = 5.33; *p* = .008; training effect in group 2 *t*(56) = −4.25; *p* = .001). The additional analysis with two time points showed trends for interaction between time and group (*F =* 3.13, *p* = .087) and for a main effect of time (*F* = 3.79, *p =* .061) at T1–T2. At T2–T3, the main effect of time (*F* = 23.76, *p* < .001) and interaction with group (*F* = 4.92, *p* < .034) confirmed that training led to more pronounced gains in the second time period. Thus, the results of the local pseudoword reading test largely coincided with the SLRT-II pseudoword results.

### Phonological awareness

The linear mixed model on the rhyme subtest of the TEPHOBE yielded a significant main effect of time (*F*(2,59) = 10.69; *p* < .001) and an interaction between the factors time and group (*F*(2,59) = 8.82; *p* < .001; see group means in Table 2). Post hoc *t* tests showed a significant improvement in group 1 during their training period (*t* (59) = −3.37; *p* = .001, *p*_corr_ = .016), but not in the waiting control period, *p*_corr_ = .140). Group 2 showed no significant improvements in any time periods, *p*_corr_ > .116. For the initial sound categorization subtest, there were no significant effects of time or interaction between time and group, *ps* > .405. Regarding the BAKO subtests, the effect of time was significant in vowel substitution *(F*(2,61) = 10.65; *p* < .001), phoneme deletion (*F*(2,58) = 4.87; *p* < .011), and a trend in pseudoword segmentation (*F*(2,57) = 2.74; *p* < .072). The interaction between time and group did not reach significance in any of these measures, *ps* > .509.

### Rapid automatized naming

#### Alphanumeric RAN

**Fig. 2:**
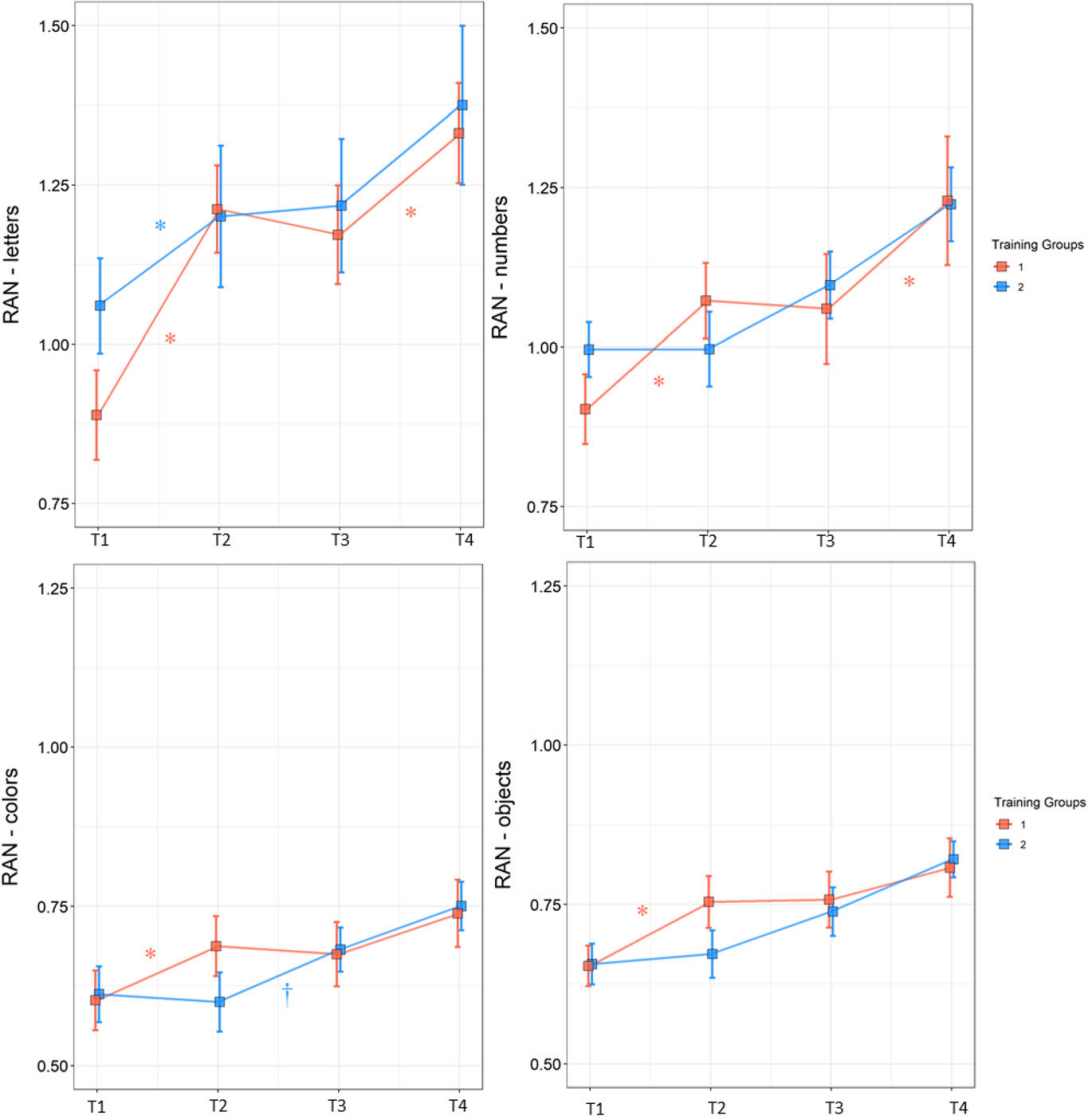
Group means in each time point for RAN subtasks. Error bars represent SEM. Data from group 1 are shown in red and data from group 2 in blue. Effects of the *t* tests between each pair of time points are indicated by asterisks (*p*_corr_ < 0.05) and daggers (*p*_corr_ < 0.1)

The linear mixed model analysis for RAN of letters yielded a significant main effect of time, (*F*(3,83) = 45.12; *p* < .001) and a significant interaction time by group (*F*(3,83) = 3.53; *p* = .018; see Fig. 2). Post hoc *t* tests per group showed a significant improvement in group 1 during the training period (*t*(83) = −6.67; *p* < .001, *p*_corr_ < .001) and in the last follow-up time period between T3 and T4 (*t*(83) = −4.07; *p* < .001, *p*_corr_ = .003), but not in the T2–T3 period, *p*_corr_ = .688. Group 2 showed significant gains in the initial waiting control period (*t*(83) = –3.45; *p* < .001, *p*_corr_ = .019) and no significant improvement in the training period or afterwards *ps*_corr_ = .534. Additional tests with two time points revealed a significant interaction time by group at T1–T2 (*F* = 4.73, *p* = .038), suggested that although there was overall improvement in both groups (main effect of time; *F* = 57.03, *p <* 0.001) group 1, receiving training, showed larger improvement than the waiting control group 2. In the second training period, there was no overall effect of time (*p* = .886) but a significant interaction between group and time *F* = 5.22, *p* = .030).

Regarding RAN of numbers, the analysis showed a significant effect of time (*F*(3,88) = 31.23; *p* < .0001) and interaction of time by group (*F*(3,88) = 2.98; *p* = .035; Fig. 2). The *t* tests per group indicated a significant improvement of group 1 during their training period, i.e., T1 and T2 (*t*(88) = −3.85; *p* < .001, *p*_corr_ = .005) and between the follow-up time points T3–T4 (*t*(88) = −5.52; *p* < .001, *p*_corr_ < .001), but not between T2 and T3, *p*_corr_ = 1. Improvements in training group 2, on the other hand, were not statistically significant in any of the tested periods, *ps*_corr_ > .636. The training effect in the first period was further confirmed in the tests with two time points. The interaction time by group was significant at T1–T2, *F* = 5.18, *p* = .030, indicated improvements in group 1 compared to their waiting controls, but there were not statistically significant main effects or interactions in the second period *ps* > .110.

#### Non-alphanumeric RAN

The linear mixed model analysis for RAN of colors with the self-developed color chart revealed a significant effect of time (*F*(3,92) = 15.81; *p* < .001) and an interaction between group and time (*F*(3,92) = 3.06; *p* = .003; Fig. 2). Post hoc *t* tests per group revealed significant improvement in group 1 during training (*t*(92) = −3.34; *p* = .001, *p*_corr_ = .025) and no improvements afterwards, *ps*_corr_ = .812. Training group 2 improvements after training approached trend levels (*t*(92) = −2.79; *p* = .006, *p*_corr_ = .111) but no significant gains in the other periods, *ps*_corr_ = .334. Additional tests with two time points revealed a significant interaction between time and group at T1–T2 (*F* = 10.56, *p* = .030) corroborating the positive training effect in the first time period. This interaction only reached trend levels in the second time period (*p =* .089).

Here, it should be noted that in the analysis using the original chart of TEPHOBE RAN of colors, the main effect of time was significant (*F*(3,89) = 11.15; *p* < .001), but the interaction between time and group only reached statistical trend levels (*F*(3,89) = 2.52; *p* < .063).

In the TEPHOBE RAN of objects, there was a significant effect of time (*F*(3,93) = 21.23; *p* < .001) and a trend for an interaction between group and time (*F*(3,93) = 2.53; *p* = .062; Fig. 2). The *t* tests in group 1 indicated a significant improvement after training (*t*(92) = −3.97; *p* < .001, *p*_corr_ = .003) and no significant improvements in the subsequent T2–T3 period or in the follow-up period, *ps*_corr_ = .116. In group 2, no difference between time points reached statistical significance, *ps*_corr_ = .198. The additional analysis with two time points revealed a significant effect of time at T1–T2, (*F* = 11.33, *p* = .002) but the interaction time by the group did not reach statistical significance (*p* = .116). At T2–T3, there was significant interaction time by group (*F* = 10.02, *p* = .003) but no main effect of time (*p* = .499).

## Discussion

This study aimed at testing whether training with GraphoLearn could improve the reading performance of below-average readers at risk of dyslexia during first and second grade. This game is designed to be a supportive training platform for children with different levels of reading abilities and may be especially useful to prevent later impairments in populations at risk. We assessed different cognitive skills important for reading like phonological processing, associating graphemes with phonemes, word and pseudoword decoding and general decoding automaticity. After training with the game, we found significant improvements in pseudoword reading after training, suggesting benefits on phonological decoding strategies in both groups. In addition, the training led to significant improvements in RAN not limited to alphanumeric RAN tasks, which may indicate a general effect on decoding automaticity.

### Benefits on phonological decoding: gains in pseudoword reading

Our main result in the analysis of SLRT-II scores indicates that improvements in pseudoword reading were aided by GraphoLearn training in both groups. In the initial period (T1–T2), only group 1, which trained in that period, showed significant improvements in pseudoword reading fluency and more children from that group improved as compared with when they received only standard school instruction. The effect sizes confirm that the improvement was larger after training. Group 1 also improved in pseudoword reading in the period following the training; however, the current data does not allow assessing whether this is a lasting training effect or whether it is due to school instruction. GraphoLearn may facilitate later benefits from school practice. It should be noted, however, that our additional analysis with two time points only supported the training effects at the second time period, that is, the training-specific improvements at T2 to T3. It is possible that differences in attainment of more basic skills between the first and the second training periods may have confounded the training effects in group 1 (see discussion on letter knowledge in the next paragraph). The SLRT-II results were supported by our local pseudoword reading test when we consider the comparable analyses including three test times. Group 2 showed statistically significant effects and group 1 a trend for improved pseudoword reading accuracy in their respective training periods. The finding in group 2 was supported by the analysis of group and time interaction at the training periods. This result is relevant because the design of the SLRT-II and the local pseudoword lists differ in terms of item complexity and measure. The former includes items of increasing difficulty and focuses on speed, while the latter includes items of mixed complexity that led to larger individual variability in scores based on reading accuracy.

Although the results in pseudoword reading suggest improved and faster phonological decoding, we found no clear training effects on the measures of phonological awareness overall. However, those measures address basic processes that largely depend on letter knowledge and grapheme-phoneme learning which is most intensively trained during the early period between T1 and T2. Thus, we could expect the groups to differ in letter knowledge, due to school instruction, at the time children began the training. Importantly, our results indicate that GraphoLearn may have a larger influence on utilizing phoneme segmentation and letter knowledge for phonological decoding of more complex items, such as pseudowords or low-frequency words which are not available in the mental lexicon.

The training strongly focuses on grapheme-phoneme mapping automaticity and it is thus likely to primarily impact the phonological decoding processes involved in pseudoword reading. Unlike ‘real’ words, pseudowords by definition do not exist in the mental lexicon and thus they must be read using an indirect, slower route of individual grapheme to phoneme conversion. The present finding is in accordance with a previous report on the English GraphoLearn (Kyle et al., 2013). That study found a positive training effect in pseudoword reading in 6–7-year-old poor readers, and the effect was stronger than that for word reading (Kyle et al., 2013). Another study found similar training effects in a Finnish sample for pseudoword reading and letter knowledge (Richardson & Lyytinen, 2014).

Regarding word reading, the results do not suggest training-related gains but rather a steady and moderate increase for most children. Here, we should consider that the current time range is a crucial period in reading development. While accuracy can reach ceiling levels in the first 2 years of instruction, word reading speed is expected to keep increasing over the years (Vaessen & Blomert, 2010; Wimmer & Hummer, 2008). The stronger improvement in word reading compared with pseudoword reading, also suggests that in this early stage of acquisition children show more difficulties with fast phonological decoding of unknown items than with expanding the mental lexicon and retrieving frequent words from it.

### Benefits on automaticity: gains in RAN skills

The observed training-related improvements in all RAN measures suggest a training effect generalized to processing automaticity. For RAN of letters, the improvement during the training period was significant in group 1, but not in group 2. This may relate to letter knowledge differences between the groups, supported by the fact that both showed gains in RAN of letters in the first period. The results also show that training offers partial support to school instruction at initial time points, as there were stronger gains in group 1 between T1 and T2, compared with group 2 (note that the T1 group differences did not reach statistical significance).

The positive training effects were more clear in the RAN of numbers. Similar results, although less pronounced, were found in the RAN of objects and colors. Altogether, they indicate that automaticity of processing was positively influenced by the GraphoLearn training. RAN skills involve multiple cognitive components: attention, visual processes of discrimination and letter identification, integration of orthographic and phonological representations, lexical retrieval and articulatory output organization (Wolf, Bowers, & Biddle, 2000). Their significance in relation to reading ability has been highlighted by a meta-analysis by Araujo and colleagues (Araujo et al., 2015). Accordingly, the evidence converges in showing a relation between RAN and reading abilities, which seems to be stronger in earlier stages of reading development and for fluency rather than accuracy measures (Wimmer, Mayringer, & Landerl, 2000).

To complement this discussion, we performed post hoc correlations and found that T1 RAN for letters correlated with improvements in word reading from T1 to T2, that is, during the training period in group 1 (*R* = 0.48, *p* = .043) and during the waiting control period in group 2 (*R* = 0.65, *p* = .012). Similar correlations were found for T1 RAN of colors which correlated with word reading gains from T1 to T2 in group 1 (*R* = 0.51, *p* = .030) and group 2 (*R* = 0.66, *p* = .007). Initial RAN did not correlate with word reading improvements in later periods. These correlational results of initial RAN for letters would be in line with the idea that letter knowledge, still under development in the first time points, may have influenced our reading fluency results and further supports the evidence on the relevance of RAN skills to reading acquisition and poor reading (Kirby, Georgiou, Martinussen, & Parrila, 2010; Moll et al., 2014). But the correlational results of initial RAN for colors suggest that more general automaticity components may be likewise relevant at early stages of word identification fluency. In this line, a recent report found RAN (average score of digits and colors) to be a consistent predictor of reading fluency from grade 1 to end of grade 2 across five alphabetic orthographies of varying complexity, including German (Landerl et al., 2019). Of note, gains in pseudoword reading in our study were not significantly correlated with T1 RAN skills, in line with the idea that pseudoword decoding relies on different processes than word identification. Finally, our finding of moderate gains in RAN, especially for letters and numbers, that continue during the follow-up period (T3-T4) could be reflecting additional school practice in second grade. We discuss the expected amount of practice and exposure in the following section.

### Limitations and implications for future GraphoLearn versions

The small sample of this study does not allow detailed analyses of individual profiles or subtypes of below-average readers at risk. However, we should emphasize that GraphoLearn is not intended as a clinical tool but as a supportive training that can help a broad population of children to improve reading by supplementing their school practice. In addition, an optimal design to assess training effects could include a larger pool of participants that were trained at the same school period to be compared with a control group without training, since it is clear that the school period in which the training starts can have an influence in the results. Although we acknowledge this limitation, our design offering training at different periods also provides an interesting window for examining individual variability in responsiveness at different stages. This is relevant as the GraphoLearn tool is intended to be useful throughout a broad school period and in parallel to school instruction. Regarding the specificity of results, it would be interesting to examine which elements of the software improve RAN skills and which are responsible for phonological decoding gains. Although this would be possible with a more restricted experimental training, in a computer-game context, it may be difficult to isolate different processes while maintaining an engaging and motivating environment. In view of our results on RAN, it would also be interesting to evaluate whether GraphoLearn may also benefit skills not limited to the language domain (e.g., visual-spatial attention, working memory). The current platform should allow for new features and tasks without excessively deviating from the main linguistic concept. Additionally, future versions would benefit from implementing more adaptive algorithms to adjust contents to individual performance profiles. A recent study on the Norwegian version of GraphoLearn suggested that including adaptive features vs fixed levels did not significantly improve the game outcomes (Solheim, Frijters, Lundetræ, & Uppstad, 2018). However, the school-based setting in that study restricted the amount of time participants could play based on their engagement. The authors also point out that the reward system should be improved to maintain engagement after the initial sessions and to optimize the level of challenge for each player.

Moreover, the game ‘timeline’ structure may benefit from an even earlier incorporation of higher level items (e.g., whole words). In the current game structure, the levels completed by the participants may not sufficiently train the automatization of whole word recognition. That is, the early levels may not sufficiently allow for transfer effects of training bigrams or single grapheme to phoneme correspondences into facilitation of sight word reading. A related limitation is the training duration. Children trained for around 11 h, similar to a previous evaluation of the English GraphoLearn (Kyle et al., 2013) and considerably longer than in two previous reports on the GraphoLearn in French (Ruiz et al., 2017) and Finnish (Hintikka, Aro, & Lyytinen, 2005), with trainings under 4 h. However, it may still be too short to yield strong and long-term benefits for reading. As revealed by a meta-analysis of intervention studies using phonics instruction, longer durations yielded considerably stronger effect sizes, for instance, trainings between 15 and 34 h compared with durations under 12 h (Galuschka et al., 2014). Finally, as mentioned in the introduction, orthographic transparency plays an important role in these differences. For instance, skills like RAN may relate to more universal mechanisms while the relative importance of phonological trainings may vary more with orthographic complexity (Landerl et al., 2019). Reading accuracy and pseudoword decoding, on the other hand, develop faster in transparent orthographies (Caravolas, 2018). Thus, adaptations of the game timeline structure, as well as duration and intensity of the training with the game should account for differences in speed of acquisition across languages.

## Conclusions

To sum up, the present Swiss German version of GraphoLearn mainly led to improvements in phonological decoding as shown by an increase in the speed and accuracy of synthetic letter-by-letter reading of pseudowords. This is one of the key abilities for reading as it supports learning of new words and progressive attainment of fluent reading skills by facilitating eventual fast word recognition by sight. We also found improvements in rapid access to stored verbal representations, i.e., rapid automated naming skills, which are strong predictors of reading development. Future versions of the training, besides emphasizing automaticity in grapheme-phoneme correspondences as in the current game, will also aim to improve sight word reading by introducing more high-frequency words of increasing complexity. Additional training forms focusing on reading for meaning will also be implemented. Our findings, after a short period of training, support GraphoLearn as a promising supportive tool for German-speaking children with reading difficulties. The results of this study can only generalize to a certain extent to a group of at risk children. Therefore, prospective studies should extend these finding to different reader profiles and to clinical populations (e.g., children diagnosed with dyslexia) to assess whether GraphoLearn may also support specialized interventions.

## Supplementary information

**Additional file 1.** Supplementary table and figures.

## Data Availability

The data that support the findings of this study are available from the corresponding author upon request. The data are not publicly available due to the restricted consent of participants. The artificial-letter training was developed at the Department of Child and Adolescent Psychiatry and Psychotherapy, Psychiatric Hospital, University of Zurich using the GraphoGame platform provided by the University Jyväskylä and is subjected to copyright.

## Abbreviations

ARHQ: Adult Reading History Questionnaire
BAKO: Basiskompetenzen für Lese-Rechtschreibleistungen
CFT: Cultural fair intelligence test
RAN: Rapid automatized naming
SLRT: Salzburger Lese- und Rechtschreibtest
TEPHOEBE: Test zur Erfassung der phonologischen Bewusstheit und der Benennungsgeschwindigkeit

## Footnotes

1 Before the second training phase, 4 subjects initially assigned to group 2 were reassigned to the group of good readers at T2 (because they unexpectedly improved reading performance) or were excluded from analyses, because they opt out of the training. 2 subjects initially assigned to the good readers group were reassigned to training group 2, because they showed unexpectedly low reading at T2. Moreover, one below-average reader joined the study at T2 and was assigned to training group 2 (final N = 16).
