# Supplementary material for: (Swiss) GraphoLearn: an app-based tool to support beginning readers
Source: Res Pract Technol Enhanc Learn. 2020 Feb 28;15(1):5. doi: 10.1186/s41039-020-0125-0 (PMC7048874; doi:10.1186/s41039-020-0125-0)
Supplement: Supplementary file 1 — Additional file 1. Supplementary table and figures. [file 41039_2020_125_MOESM1_ESM.docx]

| Table S1. Descriptive statistics and group differences in performance in GraphoLearn | | | | | | | | | |
| --- | --- | --- | --- | --- | --- | --- | --- | --- | --- |
|  |  | Group 1  n = 18 |  | Group 2  n = 16 |  | Group comparisons | | |  |
|  |  | *M* (*SD)* |  | *M* (*SD)* |  | *t* | *p-value* |  |  |
|  |  |  |  |  |  |  |  |  |  |
| Training interval duration (days) |  | 94.72 (6.39) |  | 106.56 (15.29) |  | -2.88 | .009 |  |  |
| Exposure time (minutes) |  | 638.28 ( 292.87) |  | 760.06 (214.96) |  | -1.37 | .181 |  |  |
| Number of levels |  | 259.50 (109.19) |  | 368.94 (114.32) |  | -2.85 | .008 |  |  |
| Working speed |  | 0.425 (0.12) |  | 0.4969 ( 0.12) |  | -1.75 | .090 |  |  |
|  |  |  |  |  |  |  |  |  |  |
|  |  |  |  |  |  |  |  |  |  |


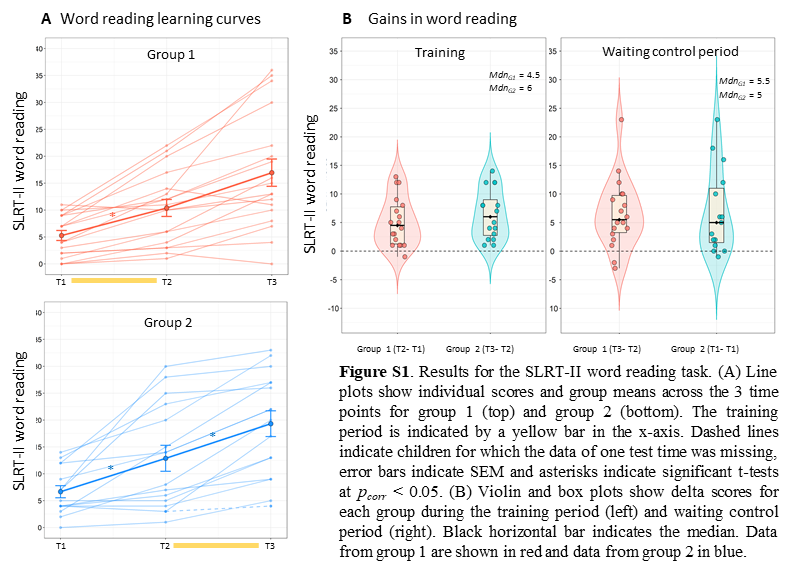


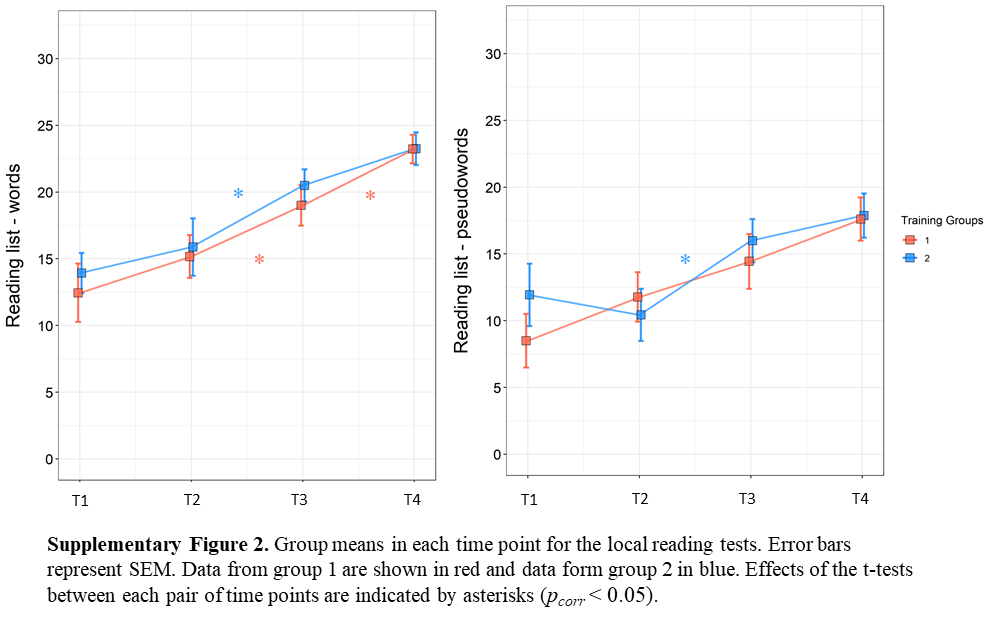


**Figure S2.** Group means in each time point for the local reading tests. Error bars represent SEM. Data from group 1 are shown in red and data form group 2 in blue. Effects of the t-tests between each pair of time points are indicated by asterisks (*p_corr_* < 0.05).
